# An Automatic Premature Ventricular Contraction Recognition System Based on Imbalanced Dataset and Pre-Trained Residual Network Using Transfer Learning on ECG Signal

**DOI:** 10.3390/diagnostics13010087

**Published:** 2022-12-28

**Authors:** Hadaate Ullah, Md Belal Bin Heyat, Faijan Akhtar, Abdullah Y. Muaad, Chiagoziem C. Ukwuoma, Muhammad Bilal, Mahdi H. Miraz, Mohammad Arif Sobhan Bhuiyan, Kaishun Wu, Robertas Damaševičius, Taisong Pan, Min Gao, Yuan Lin, Dakun Lai

**Affiliations:** 1 State Key Laboratory of Electronic Thin Films and Integrated Devices, School of Materials and Energy, University of Electronic Science and Technology of China, Chengdu 610054, China; 2 IoT Research Center, College of Computer Science and Software Engineering, Shenzhen University, Shenzhen 518060, China; 3 School of Computer Science and Engineering, University of Electronic Science and Technology of China, Chengdu 610054, China; 4 IT Department, Sana’a Community College, Sana’a 5695, Yemen; 5 School of Information and Software Engineering, University of Electronic Science and Technology of China, Chengdu 610054, China; 6 College of Pharmacy, Liaquat University of Medical and Health Sciences, Jamshoro 76090, Pakistan; 7 School of Computing and Data Science, Xiamen University Malaysia, Bandar Sunsuria, Sepang 43900, Malaysia; 8 School of Computing, Glyndŵr University, Wrexham LL11 2AW, UK; 9 Department of Software Engineering, Kaunas University of Technology, 44249 Kaunas, Lithuania; 10 Medico-Engineering Corporation on Applied Medicine Research Center, University of Electronic Science and Technology of China, Chengdu 610054, China; 11 Biomedical Imaging and Electrophysiology Laboratory, School of Electronic Science and Engineering, University of Electronic Science and Technology of China, Chengdu 610054, China

**Keywords:** premature ventricular contraction, electrocardiogram, recognition, transfer learning, imbalanced datasets, patient-specific, residual network, pre-trained

## Abstract

The development of automatic monitoring and diagnosis systems for cardiac patients over the internet has been facilitated by recent advancements in wearable sensor devices from electrocardiographs (ECGs), which need the use of patient-specific approaches. Premature ventricular contraction (PVC) is a common chronic cardiovascular disease that can cause conditions that are potentially fatal. Therefore, for the diagnosis of likely heart failure, precise PVC detection from ECGs is crucial. In the clinical settings, cardiologists typically employ long-term ECGs as a tool to identify PVCs, where a cardiologist must put in a lot of time and effort to appropriately assess the long-term ECGs which is time consuming and cumbersome. By addressing these issues, we have investigated a deep learning method with a pre-trained deep residual network, ResNet-18, to identify PVCs automatically using transfer learning mechanism. Herein, features are extracted by the inner layers of the network automatically compared to hand-crafted feature extraction methods. Transfer learning mechanism handles the difficulties of required large volume of training data for a deep model. The pre-trained model is evaluated on the Massachusetts Institute of Technology-Beth Israel Hospital (MIT-BIH) Arrhythmia and Institute of Cardiological Technics (INCART) datasets. First, we used the Pan–Tompkins algorithm to segment 44,103 normal and 6423 PVC beats, as well as 106,239 normal and 9987 PVC beats from the MIT-BIH Arrhythmia and IN-CART datasets, respectively. The pre-trained model employed the segmented beats as input after being converted into 2D (two-dimensional) images. The method is optimized with the using of weighted random samples, on-the-fly augmentation, Adam optimizer, and call back feature. The results from the proposed method demonstrate the satisfactory findings without the using of any complex pre-processing and feature extraction technique as well as design complexity of model. Using LOSOCV (leave one subject out cross-validation), the received accuracies on MIT-BIH and INCART are 99.93% and 99.77%, respectively, suppressing the state-of-the-art methods for PVC recognition on unseen data. This demonstrates the efficacy and generalizability of the proposed method on the imbalanced datasets. Due to the absence of device-specific (patient-specific) information at the evaluating stage on the target datasets in this study, the method might be used as a general approach to handle the situations in which ECG signals are obtained from different patients utilizing a variety of smart sensor devices.

## 1. Introduction

ECG is a popular non-invasive method commonly employed to diagnose heart [1,2,3,4] abnormalities such as arrhythmias. While some forms of arrhythmias can be harmless, some others are life-threatening. Life-threatening arrhythmias involve tachycardia and ventricular fibrillation and demand immediate treatment. Premature ventricular contraction (PVC) requires prompt treatment to confine the escalating health complexities. PVC is comparatively less harmful than life-threatening arrhythmias. However, it is still a matter of concern as repeated occurrences may increase heart disorders and rise in total mortality in several subgroups of patients [5]. Therefore, further research and development are needed for rapid, accurate, and automatic diagnosis of PVC.

Computer-aided or automatic recognition of PVC can alleviate the specialists’ workload and enhance the efficiency of diagnosis. In recent years, many approaches concerning automatic detection of PVC from ECG signals have been proposed by several researchers [6,7,8] using traditional feature engineering learning techniques [9]. Beyond the PVC diagnosis from ECG signals, there are vigorous applications of ECG in real-life applications. As for the example, one dimensional MLGLCM (multi-layer co-occurrence matrices) is used to extract the Haralick features from ECG signals for identifying the individuals, where the extracted features are feed to the classifiers such as K-nearest neighborhood (KNN), Naive Bayes (NB), random forest (RF), Bayes net (BN), support vector machine (SVM) for the recognition of personal identity [10]. Beyond the MLGLCM and the aforementioned classifiers used in traditional feature learning approaches, there are various feature-extraction and selection techniques such as wavelet transforms (WTs), discrete wavelet transform (DWT), discrete cosine transform (DCT), empirical mode decomposition (EMD), principal component analysis (PCA), independent component analysis (ICA), Linear discriminant analysis (LDA) and classifiers such as artificial neural networks (ANN), AdaBoost (AB), decision tree (DT), logistic regression (LR) [11]. Zhao et al. [12] developed a method to identify the PVCs from RVOT (right ventricular outflow tract) and LVOT (left ventricular outflow tract) of surface ECGs characteristics using RF classifier and achieved the satisfactory results. Sraitih et al. [13] investigated four machine learning approaches with SVM, KNN, RF, and ensemble of these three methods to classify the PVC (premature ventricular contraction), RBBB (right bundle branch block beat), LBBB (left bundle branch block beat), PAC (premature atrial contraction), and NOR (normal beat) in inter-patient paradigm mechanism. Their findings demonstrated that the results with SVM outperform the other approaches. PVCs could also be detected from the photoplethysmography (PPG) signals [14].

However, with the recent development of various deep learning techniques [15,16,17,18], deep learning can now provide better automatic learning of the desired features from the input (i.e., raw ECG signals) compared to the feature engineering techniques [19]. Deep learning-based approaches, such as convolution neural networks (CNNs) [20,21,22,23], deep neural networks (DNNs) [24,25], recurrent neural networks (RNNs), and long short-term memory (LSTM) [26,27], as well as assembling of such architectures [28,29] could be employed for PVC diagnosis as well as in various applications with ECG. As for the examples, AKDAĞ et al. [30] proposed a deep learning approach for arrhythmia and congestive heart failure recognition based on 1D-LBP (one-dimensional local binary pattern) with downsampling and LSTM, where the link among the neighbors’ smallness and bigness is processed using 1D-LBP and histogram features (obtained with downsampling the signal) of 1D-LBP of various signal groups are used as the reference of LSTM. Their experimental results demonstrated that the diagnostic performance accuracies vary from 96.80% to 99.79% in various scenarios. Angle transform (AT) approach with LSTM could also perform the same task described in [30] with satisfactory results [31]. With the growing interest of biometric authentication, deep learning plays an important role as a reliable tool to the function easily and accurately from ECGs [32]. Searching of PVCs from the time-series ECG signals with 1D CNNs is a common detection approach and provides good findings in some scenarios [33]. Herein, features are extracted automatically compared to hand-crafted feature extraction methods discussed aforementioned and a Softmax classifier is usually used for the classification in the last layer. Sometimes, well-known classifiers such as KNN, RF, SVM, ANN are used to detect or classify the PVCs utilizing the deep learning metrics extracted with a deep model [34]. Nowadays, 2D CNNs are more promising due to their better visual perception for diagnosing cardiac diseases [16,35] as well as reducing the strict time alignment requirement of time-series data as in the 1D CNNs approaches.

The 2D CNN-based model, ResNet [36], is a significant development in deep learning. It has a shortcut connection between the back and the front layers, which helps to reduce the vanishing gradient problem during training. As a result, deeper CNN can better manage the big data from various real-life applications. Considering the significant potentials of 2D CNNs and ResNet networks, we proposed a method to classify the patient-specific PVCs with a pre-trained ResNet (ResNet-18) using a transfer learning mechanism on the pre-processed images from two publicly available imbalanced datasets, namely MIT-BIH Arrhythmia and INCART. Class imbalance is a common challenge in real-life applications to recognize a specific disease. The imbalanced class datasets lead the machine learning algorithms towards a high misclassification rate, negatively impacting results [37]. Therefore, despite contriving a satisfactory approach to resolve the issues in morphological-based machine learning algorithm, an end-to-end deep learning method is required to correctly identify the PVCs in real-life applications [38].

Deep learning-based methods demand a large volume of training data for addressing the challenge of recognizing all the desired patterns from the ECG signals [39,40,41,42], where some patterns are even difficult to be recognized by experienced cardiologists [25]. However, the private and publicly available datasets usually contain a small volume of data which strongly opposes the comprehensive use of DNNs. The transfer learning strategy could resolve the challenge, where an upstream (large) dataset is transferred to a downstream (target-usually small) dataset. The transfer learning mechanism alleviates the over-fitting of a deep model. It provides high credibility to recognize the abnormalities from the target domain [43]. Therefore, the transfer learning technique can effectively be used in resource constraint devices, where the testing model is pre-trained with a large volume of data so that no more storage space and data to train the model are required to receive a service. A significant number of wearable ECG monitoring devices have recently emerged, for the example [44], which provides a large volume of patient-specific data. This trend of wearable devices imposes the need for developing data-driven and true generic device-independent (patient-specific) methods for ECG signal analysis. Recently flexible and stretchable wearable sensors play a great role in this regard with the emerging of different flexible materials as well as designing strategies of sensors [45]. Implementing such a strategy, numerous efforts, for example [46] have been observed to classify the abnormality of ECG beats using different methods. Though the present attempts contribute to the consolidated directions of this domain, the device-independent problem has yet been properly addressed. This is due to high morphological features variation of ECG signals from different patients and various hardware implementations in different devices.

Moreover, the recent work of Kiranyaz et al. [47] summarized two major problems in the existing patient-specific methods: (1) a single classifier cannot guarantee accurate identification of a new patient and (2) earlier knowledge of a patient on both beats (normal and abnormal) is required. However, effective automatic detection of heart rhythm or beats is still challenging due to the observable variations of temporal and morphological features, such as QRS complex shapes, RR intervals and P waves of heart patients under various physical and temporal situations. Considering the potential benefits of (i) the rigorous time alignment requirement is lessened when time-series data str transformed into their corresponding images during features extraction, (ii) 2D approach provides better visual perception at some scenarios in clinics, (iii) shortcut connection reduces vanishing gradient problem during training when network goes to deeper, (iv) using of several diverse techniques such as early stopping [48], weighted random sampler [49], Adam optimizer [50], (v) Pan–Tompkins algorithm (an effective beat segmentation algorithm) [51], (vi) patient-specific evaluation technique (more reliable approach for real-life applications [52]) which reduces the biasing of results, and (vii) transfer earning mechanism for the small volume of target datasets, we have proposed a deep learning method based on 2D CNN with a pre-trained deep residual network, ResNet-18, to identify PVCs automatically using transfer learning mechanism in this study. To the best of our knowledge, this is the first attempt to identify the PVCs with a 2D CNN based method. Firstly, we have segmented PVCs and normal beats from a benchmark dataset (MIT-BIH arrhythmia) and INCART dataset (for the generalizing purpose of the proposed method) using the Pan–Tompkins algorithm [51] and then transformed them into beat images. The ResNet is pre-trained using the large volume dataset named ImageNet, so that the networks are properly trained and can effectively retrieve complex patterns of objects from the extracted beat images. The knowledge from ImageNet is transferred to the target domain of small volume datasets (MIT-BIH and INCART)) for recognizing the PVC from the normal beats. The pre-trained ResNet-18 is fine-tuned with the extracted PVC and normal beat images and provides satisfactory results using a patient-specific mechanism. The pre-training helps to easily track the weights learning from the target (downstream) dataset. Herein, the pre-trained ResNet-18 performs as the feature extractor for PVC identification. The study aims to verify an end-to-end deep learning method without utilizing any complicated pre-processing and feature extraction techniques on the raw information, keeping the model’s design very simple.

The major contributions of the study are:

- Identify PVCs of unknown patients using patient-specific classification strategy.
- In most cases, the proposed method suppresses the performance of state-of-the-art methods on imbalanced datasets.
- Efficient implementation of several diverse regularization techniques in the proposed method, such as call-back features [48], weighted random sampler [49], Adam optimizer [50] and on-the-fly augmentation of data [53] resolve the device-independent issues.

The remainder of the study is presented as follows. In Section 2, the proposed method and materials are given. In Section 3, experimental results and discussion with related works on PVC detection are presented following the findings of the proposed methodology in Section 2. Finally, the study is concluded with several future directions.

## 2. Methods & Materials

### 2.1. Method Overview

**Figure 1:**
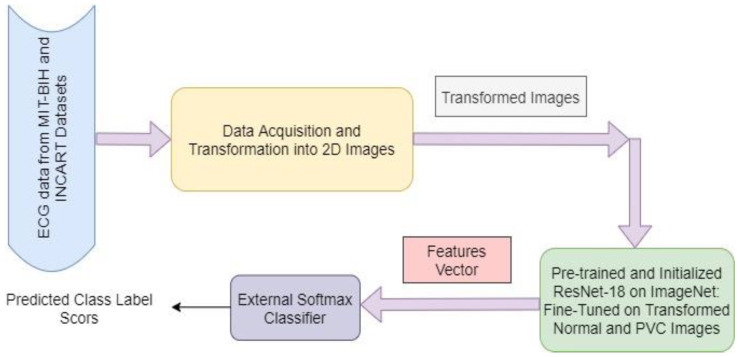
The proposed method overview using transfer learning on imbalanced datasets.

Our proposed method involves a pre-trained model (ResNet-18) that is trained on a large volume dataset (ImageNet), and learned knowledge from ImageNet is transferred into a small volume of datasets (target datasets- MIT-BIH and INCART). First, annotated data are received from the MIT-BIH and INCART 12-lead Arrhythmia Datasets. Then every data instance of normal and PVC beats from these datasets is transformed into the corresponding 2D beat images through the pre-processing step. The transformed images are used as the inputs of the pre-trained model. The extracted generic descriptors from the inside layers of ResNet represent the existence of different categories of image patterns. This learning experience of ResNet on the ImageNet dataset is transferred into the proposed method to recognise the PVCs from normal beat images. The classification layer of ResNet is replaced with a fully connected (FC) layer along with a Softmax classifier for fine-tuning, where the weights of the FC layer are randomly initialized. However, the class numbers remain identical to the target classes. The proposed method with transfer learning is demonstrated in Figure 1. We have performed mainly four cross-validation strategies (experiments), i.e., T1 (MIT-BIH training and INCART testing), T2 (INCART training and MIT-BIH testing), T3 (LOSOCV-leave one subject out cross-validation on MIT-BIH) and T4 (LOSOCV on INCART), on the patient-specific pattern to evaluate the performance of our proposed method. It demonstrated that the models trained and evaluated with the same patients are biased, and the results are not replicated for real-life applications [52].

So, we have chosen a patient-specific paradigm in our study, where independent training and testing sets are considered in T1 and T2 from different patients as well as different datasets, while the subjects are varied in every fold of the cross-validation process during the training and evaluation in T3 and T4 for each dataset. The patient-specific mechanism has a good predictive ability. It is efficient when run on resource constraint devices for real-life applications.

### 2.2. Dataset Details and Data Collection

MIT-BIH arrhythmia database: It includes a total of 48 records from 47 subjects. The records were sampled at 360 Hz per channel with 11-bit resolution over 10 mV and annotated independently with two or more cardiologists. The dataset has two channels (a modified limb lead II (MLII) and modified lead V1). In this study, MLII is chosen to collect the ECG data because QRS complexes of normal waves are more observable in this lead. Records 102, 104, 107 and 217 are ignored in our experiments due to the involvement of paced beats and surgical dressings of patients. Among the remaining 44, we have used only 22 records, which involve the PVC beats.

INCART 12-lead Arrhythmia Database: INCART dataset holds a total of 75 annotated recordings, where each record is 30 min long and contains 12 lead information. The records were sampled at 257 Hz per channel with varying gains from 250–1100 per mV. The records were collected from patients who suffered from various cardiovascular diseases. Among the 12 standard leads of the dataset, QRS complexes are more remarkable in the lead named ‘lead II’. Hence, we have chosen lead II for our study. We have considered most of the records (61) amongst the 75 involving PVC and other categories of beats. However, in experiment T4, we have considered only 22 records out of 61.

### 2.3. Data Pre-Processing

Firstly, the annotation files from each record of both datasets (MIT-BIH and INCART) were received by the python Glob module and processed with WFDB Toolbox and Biosppy module to segment the available normal and PVC beats in a record. The R-peaks detection from the QRS-complexes is performed with the Pan–Tompkins algorithm [51] with a sampling frequency of 200 Hz. After detecting R-peaks, a single beat is counted by any R-peak and its next R-peak, taking half distance of those. Matplotlib and OpenCV python modules transform the segmented beats into the equivalent RGB images. Herein, time-series ECG signals are converted into 2D RGB images, which are fed as the input of the pre-trained ResNet-18 to extract the desired features from normal and PVC beat images. A high-level feature vector is constructed from these features, and finally, PVC is identified based on that vector through a Softmax classifier.

### 2.4. ResNets/Residual Networks

Deep CNNs usually integrate the low-level features and classifiers with an end-to-end multilayer structure, where feature levels could be enhanced by stacking layers (depth). This structure of CNNs leads it to a sequence of successes in image classification [54]. However, the recent experiments on a large dataset (ImageNet) demonstrate that deeper networks are subject to the exploding/ vanishing gradient problem [55]. This problem adversely impacts the convergence of the network from the beginning and degrades the performance. He et al. [36] addressed the problem by commencing a deep framework with a residual/identity mapping, namely residual network (ResNet).

**Figure 2:**
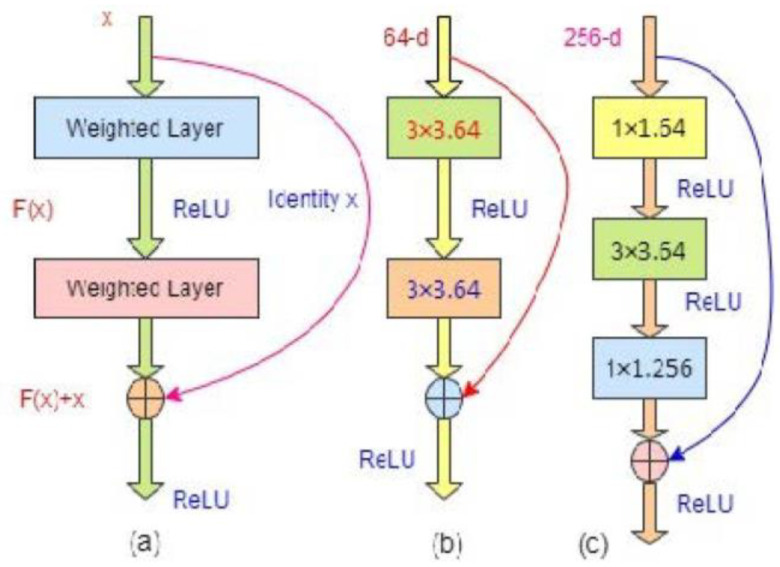
(**a**) The basic building block structure of residual network; (**b**) A building block for ResNet-18/34; (**c**) A building block for ResNet-50/101/152 (bottleneck structure).

In the residual network, original/underlying mapping, H(x) (which is fitted with a few stacked layers) is reformatted into F(x)+x, where F(x):=H(x)−x, x denotes the input in the first of these layers and F(x) is the residual function (where input and output of the function are of identical dimensions). This formation of F(x)+x is realized in the residual network with a shortcut connection depicted in Figure 2, where one layer is skipped. The shortcut connection mainly carries out the identity mapping [56] that adds neither extra computational complexity nor learnable parameters. A residual building block with the identity mapping can be defined as follows:

(1) $$y=F\left(x,\left\{W_{i}\right\}\right)+x$$

where y and x are the output and input vectors of the considered layers, and $F\left(x;\left\{W_{i}\right\}\right)$ expresses the residual/identity mapping to be experienced/learned.

In Figure 2a, the block has two layers and follows: $F=W_{2}\sigma \left(W_{1}x\right)$, where σ represents the activation function, rectified linear function (ReLU) [57]. The biasing is ignored to simplify the notations. Usually all learnt abstract representations in deep learning are independent of one another, which is often undesirable. Therefore, non-negative activation functions are preferred for CNN. The ReLU, f(x) = argmax (0, x), is the most prevalent of these functions, and a neuron that employs it is known as ReLU. The several major benefits of ReLU over sigmoidal functions such as tanh(x) and σ (x) are: (i) it could be calculated easily because only it is required to compare its input to the value of 0, (ii) depending on whether or not its input is negative, it has a derivative of either 0 or 1, and (iii) it has great implications in back propagation for training a network which expresses the computing of gradient of a neuron is not so expensive. ReLU (x) is equal to 0 for x < 0, while it is equal to 1 for x ≥ 0. As a result, using ReLU lessens the likelihood that the computing needed to run the neural network would raise exponentially. The computational cost of adding additional ReLUs rises linearly as the CNN’s size scales. Moreover, the so-called “vanishing gradient” problem, which frequently occurs in deeper networks when sigmoidal functions are used, could be avoided by ReLUs. The operation (F+x) is carried out with the shortcut phenomenon and element-wise addition, where the dimensions of F and x should be equal. The second nonlinearity could be adopted by adding σ(y) in Figure 2a. If the output/input channel numbers are changed, then a linear projection, $W_{s}$ (square matrix), is included in the identity mapping by element-wise multiplying with the input channel numbers to match the dimensions as follows:

(2) $$y=F\left(x,\left\{W_{i}\right\}\right)+W_{s}x$$

Herein, the form of identity function, F, is flexible. The function involves two or three layers in Figure 2b,c, respectively, where either more or less is also acceptable; however, if it has only one layer, Equation (1) is identical to a linear layer: $y=W_{1}x+x$, without providing any significant benefit for such structure. This notation/structure is more applicable for fully connected layers, while $F(x;\;\left\{W_{i}\right\}$ is for the multiple convolutional layers. The element-wise addition is carried out by channel on two feature maps.

Figure 2b expresses the building block of ResNets-18/32, while Figure 2c for ResNet-50/101/152 holds three layers: 1 × 1, 3 × 3, and 1 × 1 convolutions. The foundation of a CNN is the convolutional layers. It has a number of kernels or filters, whose settings must be learned over the course of training. Herein, 1 × 1, 3 × 3, 1 × 1 and 64, 256 represent the kernel sizes and kernel number respectively for the assigned convolution layers in a CNN. Typically, the filters’ size should be smaller than the original images in 2D CNNs. Each filter convolves the images to produce a map of activation. For the convolution, the filter is slid over the image’s width and height, and at each spatial location, the dot product between each filter element and the input is determined. The same procedure is repeated for each component of the input image to create the activation map. The activation maps of each filter are stacked along the depth dimension to create the convolutional layer’s output volume. Every element of the activation map can be considered to be a neuron’s output. As a result, each neuron is connected to a discrete local area in the input image, and the area’s size is equal to the filter’s size. All of the neurons in an activation map share parameters as well. The convolutional layer’s local connection forces the network to learn filters that respond most strongly to a particular local region of the input. The early convolutional layers of an image capture its low-level details, such as its lines, whereas the later layers extract its high-level details, such as its forms and particular objects. The 1 × 1 layers (bottleneck layers) are liable for reducing and restoring or increasing (leaving 3 × 3 layer) the input/output dimensions to match the same dimensions in the building blocks of the network, where the projection shortcut of Equation (2) is employed. The interesting point is that both structures have the same time complexity [36].

**Figure 3:**
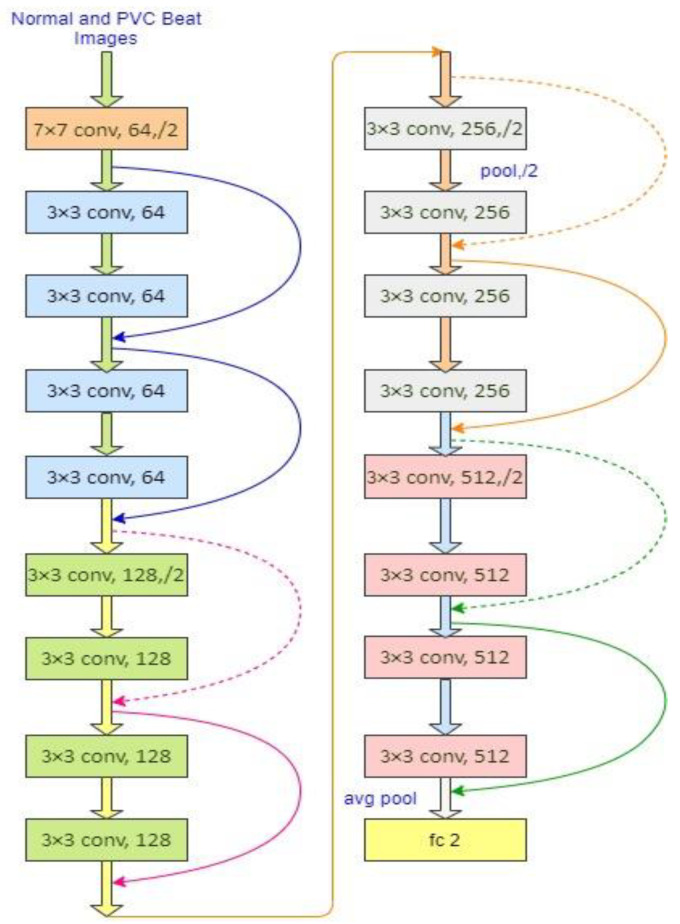
ResNet-18 network architecture, the dotted shortcuts represent the enhancement of the dimensions.

A residual network of 18 layers is illustrated in Figure 3, where a solid line represents the direct use of identity shortcuts following Equation (1) for the same dimensions of input/output in a residual block. When the dimension increases, the blocks follow the shortcut lines as the dotted line depicted in Figure 3. It is maintained in two ways: (1) the shortcut can follow the identity mapping with an additional zero entries of padding to enhance the dimension without introducing any extra parameter, and (2) the projection shortcut is employed to match the dimensions. In both cases, the shortcuts belong to the feature maps performed with the stride of 2. The number of pixels shifted across the input matrix called the stride. The filters are moved to 1 pixel at a time when the stride is 1. The filters are moved to 2 pixels at a time when the stride is 2, and so on. Convolution layers in each block extract information from the input images. With the learning image features with small squares of input data, convolution preserves the relationship between pixels by a mathematical operation with two inputs: an image matrix and a filter. The volume of an input image could be represented as: h × w × d, where h, w, d indicate the height, width, and dimension of an image respectively. Then, the output volume will be: (h − f_h_ + 1) × (w − f_w_ + 1) × 1 for the filter f_h_ × f_w_ × d. Sometimes, the filters are not able to perfectly fit the input image. In that case, we have two choices: (i) pad the image with zeros to make it fit (zero-padding), (ii) removing the portion of the image that did not fit with the filter called valid padding (it keeps only the valid parts of the image). 

In this study, option 1 is followed. When the images are too large, the pooling layers section would reduce the number of parameters. Spatial pooling, also known as subsampling or downsampling, reduces the dimensionality of each map while retaining critical information. There are several types of spatial pooling: max pooling, average pooling, and global average pooling. The largest element from the rectified feature map is used in max pooling. The largest element could also be chosen with average pooling. Moreover, global average pooling sums all of the elements in feature map. Finally, the resulted matrix is flattered into a vector and fed to the fully connected layer and employs the Softmax function to classify an image with the probabilistic values from 0 to 1. The parameter-free identity mapping (option-1) is usually essential for bottleneck architectures, as shown in ResNet-50/101/152. In all the versions (ResNet-18/34/50/101/152) of ResNet, it holds a 3 × 3 filter in the convolutional layers following mainly two design rules: (1) the layers must have the same number of filters as the output size of the feature map; (2) the filter number will be doubled when the size of feature map is halved, so that time complexity per layer is preserved. Down-sampling is performed by the convolutional layers directly with the stride of 2. The network is ended with a layer of global average pooling and a Softmax layer through a 1000-class fully connected layer in all the versions.

In our study, the network follows 2-classes fully connected layer based on the desired class number from our target datasets. ResNets [36] have lower complexity and filters than VGGNets [55]. ResNet-34 has only 3.6 billion FLOPs (floating-point operations per second), which is only 18% of VGG-19 (19.6 billion FLOPs). The details of all variants of ResNet are demonstrated in [36]. He et al. [36] performed their experiments with the ResNet-18/34 on ImageNet and a tiny image dataset CIFAR-10 set by following the phenomena of shortcut connections and observed that the network is easily optimized with fewer degradation problems as well as less training/testing errors compared to the same network of plain structure (that stacked the layers without the skip connections). They also observed that the network provides good accuracy and substantially with the increasing depth of network by the phenomena of shortcut connections. A residual network parameter with 34 layers [36] is eight times deeper than VGGNet-19 [55]. Due to the depth representation benefits, residual networks are now widely used in various visual recognition tasks such as diseases detection, traffic flow analysis and detection, security, etc.

### 2.5. Weighted Binary Cross-Entropy Loss

Cross-entropy loss is used to evaluate the degree to which a model is appropriately trained, indicating the distinction between true and predicted label scoring training loss. It reduces the gap between the targets and measured labels, greatly influenced by an optimizer with a specific learning rate.

In this study, we have used the Adam optimizer, because it usually reaches the optimal points faster than other existing ones [50]. The extracted beats show that the numbers of normal and PVC beats are significantly imbalanced. So, for every training epoch, the normal samples will have more influence than the PVC samples for updating the network parameters. This scenario will significantly undermine the network’s performance in identifying the PVCs. To address this problem, we have employed a weighted binary cross-entropy loss function which could be expressed as follows:

(3) $$l=\sum_{x\in X}\eta_{i\left(x\right)}\left[y^{true}\ln y^{out}+\left(1-y^{true}\right)\ln\left(1-y^{out}\right)\right]$$

where i(x) indicates the target label of the sample x, $y^{true}$ and $y^{out}$ represent the model ground truth and output labels, respectively. The $\eta_{i\left(x\right)}$ expresses the weight co-efficient, which can be denoted as below:

(4) $$\eta_{i\left(x\right)}=\left(1-\frac{\left|X^{i\left(x\right)}\right|}{X}\right)$$

where |Xi(x)| reveals the sample numbers belonging to the target i(x) of the training batch X. 

From Equation (3), it is observed that the classes with fewer sample numbers provide a large weight. As a result, PVC beat images equally contribute to loss with normal beat images in each of the epochs during the training.

## 3. Experimental Results and Discussions

### 3.1. Classification Results

The experiments are performed with Intel Core i5-7400 CPU (3.00 GHz), 8 GB DDR RAM, and NVIDIA GeForce RTX 2070 graphics processing unit (GPU) with 8 GB memory. PyTorch framework [58] of deep learning is chosen to implement the proposed method with NVIDIA GPUs using CUDNN and CUDA [59]. The training set in each experiment is divided into a validation portion (10%) of the whole data using a random split mechanism. The optimized initial learning rate and batch size are 0.001 and 32, respectively. Adam optimizer [50] with a PyTorch learning rate scheduler (REDUCELRONPLATEAU) is chosen to optimize the loss function. The learning rate declined by a factor of 0.1 if the validation loss lasted for five successive epochs. The weighted random sampler [49] employed in the developed training module during the experiments to ensure the equal representatives in every class of samples. In this study, we have employed early stopping regularization technique [48] to halt the training if validation loss remains the same for eight successive epochs. This feature helps the training module to achieve the optimal time for training and control the over-fitting. As for the example, the training in T_1_ halted at 67 epochs because of employing the feature, but the total epoch’s number was set to 200. The training module checked the validation loss of eight consecutive epochs. Since there was no change of validation loss from 60 to 67, it halted at 67 epochs. Moreover, we can easily change this number (eight) by any numeric number such as six, ten, twelve, or fifteen in the module. Then the system will use that number to halt the training. The performance of the proposed method is measured with the following Equations (5)–(9) [60,61,62,63]:

(5) $$precision=\frac{TP}{TP+FP}$$

(6) $$recall=\frac{TP}{TP+FN}$$

(7) $$F_{1}=2\times \frac{precision\times recall}{precision+recall}=\frac{2\times TP}{2\times TP+FP+FN}$$

(8) $$specificity=\frac{TN}{TN+FP}$$

(9) $$accuracy=\frac{TP+TN}{TP+FP+TN+TF}$$

(10) $$balanced\_accuracy=\frac{recall+specificity}{2}$$

where True Positive (*TP*) reveals positive identified as the positive, while False Negative (*FN*) expresses positive identified as the negative. In contrast, *TN* represents negative signified as the negative while *FP* indicates negative signified as positive.

The performances of the classifier and the proposed method were observed and evaluated with the loss function, evaluation metrics and confusion matrix. Table 1 illustrates the confusion matrix of the proposed method for the experiments T1, T2, T3, and T4. It is observed that only 62, 13, 04, and 29 PVC images are misclassified out of 9987, 6423, 6423, and 8316 in T1, T2, T3, and T4, respectively, while 9925, 6410, 6419, and 8287 PVC images are correctly classified in T1, T2, T3 and T4, respectively.

**Figure 4:**
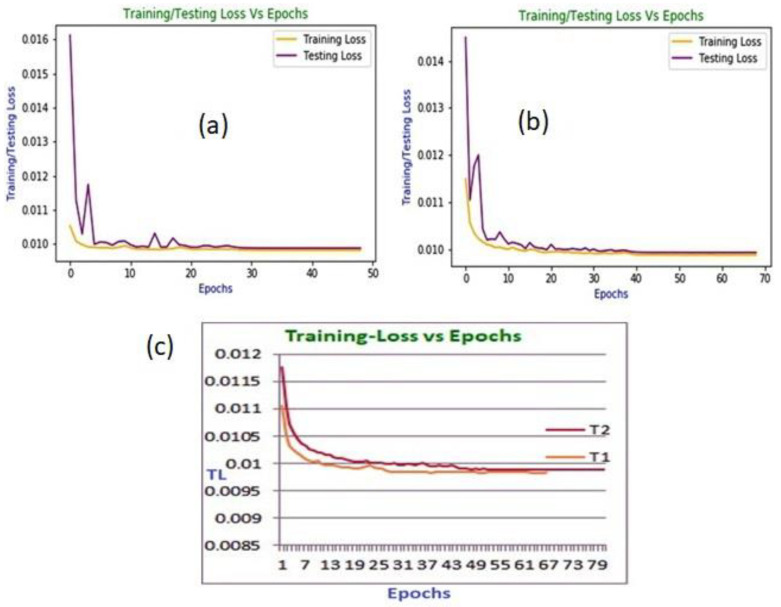
(**a**) The training and testing loss versus epoch curve in T3; (**b**) The training and testing loss versus epoch curve in T4; (**c**) The training loss versus epoch curves in T1 and T2.

The training and testing loss curves of the proposed framework in T3 and T4 are demonstrated in Figure 4a,b, respectively. These loss curves show that the training curves start to plateau after around 28 and 39 epochs in T3 and T4, respectively. The testing curves demonstrated unexpected movement at the beginning. However, they almost remained at a plateau after they reached close to the same number of epochs of training curves.

The training loss (TL) versus epoch curves for the proposed framework in T1 and T2 are demonstrated in Figure 4c, from which it is evident that the curves start to become stable after around 54 and 52 epochs in T1 and T2, respectively. Moreover, the training is halted at 49 and 69 epochs in T3 and T4, respectively, while the training is halted at 67 and 81 epochs in T1 and T2, respectively, as observed from Figure 4a–c. The pre-trained model performs training and testing (evaluation) on the transformed PVC beat images without experiencing any over-fitting. So, it attains the intended level of achievements on the MIT-BIH and INCART datasets.

**Figure 5:**
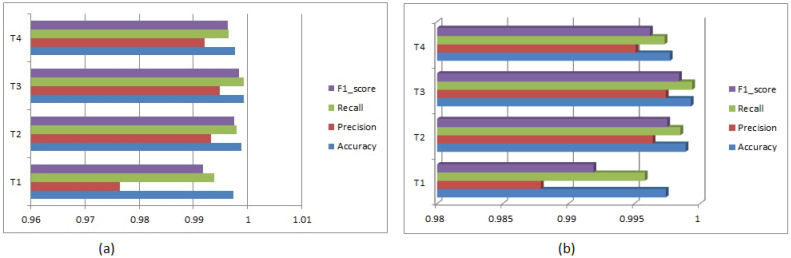
(**a**) Bar graph for the summary of all achieved metrics in all the experiments; (**b**) Bar graph for the summary of all achieved metrics from the confusion matrix in all the experiments.

A summary of received overall accuracies, precisions, recalls, and F1-scores to identify the N and PVC for all experiments is shown in Table 2 and a bar graph for these metrics is illustrated in Figure 5a. The minimal validation losses are 0.0099, 0.0098, 0.0099 and 0.0099 in T1, T2, T3, and T4, respectively, during the training. Table 3 also demonstrates a summary of obtained metrics (accuracy, precision, recall, F1-score and specificity) from the received confusion matrixes for all the experiments and a bar graph is depicted in Figure 5b. After optimizing the parameters in experiments T1 and T2 over the classifier’s training set, we evaluated the classifier with those parameters in the testing phase, where identification samples (PVC beat images) come from different individuals. However, in experiments T3 and T4, the subjects are changed in each cross-validation fold for evaluation. This provides independent subject estimation, which is essential in resource constraint devices such as mobile, wearable/portable healthcare devices. Moreover, we have evaluated balanced accuracy for all experiments using the Equation (10) and achieved 99.58%, 99.85%, 99.94%, and 99.73% for T_1_, T_2_, T_3_, and T_4_, respectively, while accuracies are 99.74%, 99.89%, 99.93%, and 99.77%. The results express that accuracy and balanced accuracy are near to being achieved. We have also tested the results from the sklearn.metrics module of Python using sklearn.metrics.accuracy_score and sklearn.metrics.balanced_accuracy_score functions and received the more close results for both metrics [64]. Sometimes, balanced accuracy is also represented as the un-weighted average recall, where summing accuracies of all classes is divided by the total number classes.

### 3.2. Comparison with the State-of-the-Art Works and Discussions

**Figure 6:**
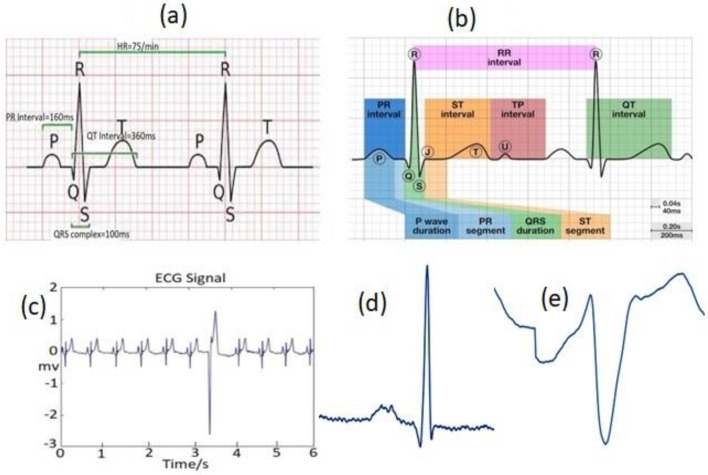
(**a**) A normal ECG signal including a typical P-QRS-T complex and RR-intervals; (**b**) A normal ECG signal with U-wave deflection; (**c**) A sample waveform of a PVC beat and normal beats from the MIT-BIH arrhythmia dataset of a record; (**d**) A sample of the extracted normal image from MIT-BIH; (**e**) A sample of the extracted PVC beat image from MIT-BIH.

The ECG waves (mainly P-trial depolarization, QRS-ventricular depolarization, and T-ventricular repolarization/relaxation), where the QRS complex is more crucial [65]. These waves, demonstrated in Figure 6a, correspond to a specific electrical phenomenon’s induced field on a cardiac surface. Each signal phase has a specific time duration/amplitude range, as stated in Table 4. ECG diagnosis usually involves prime clinical features such as QRS durations, R-R intervals, and S-T-U duration (sometimes, a small U-wave deflection (0.5 mm) appears in an ECG signal following T-wave due to the delaying repolarization of Purkinje fibers, prolonged repolarization of mid-myocardial “M-cells”, mechanical forces in the ventricular wall (as shown in Figure 6b) and P wave duration.

PVC is one of the most crucial but common arrhythmias amongst the different categories of abnormalities relating to the functioning of a human heart. It is the abnormal contraction (electrical activity) of the lower chambers of the heart (ventricles) due to the presence of ectopic centers in the heart ventricles. It usually produces QRS complexes with wide waveforms indicating possible life-threatening cardiac circumstances [66]. The T-wave usually starts after each S-wave, making the window of 60 samples (0.166 s). This wave in PVC is oriented in the reverse direction to the QRS-complex [67], as shown in Figure 6c. The figure illustrates the sample waveform of a PVC beat and normal beats from the MIT-BIH arrhythmia dataset of a record. Figure 6d,e also demonstrate the sample of the extracted normal and PVC beat images from the MIT-BIH arrhythmia dataset, respectively. Note that the characteristics of extracted beat images from INCART dataset are almost similar to the MIT-BIH arrhythmia dataset. The amplitude of the R-peak of PVC was >125% of a normal peak when an ectopic beat originated from the left ventricle. However, the R-peak amplitude of PVC is <50% when an ectopic beat originates from the right ventricle [68]. The P-wave vanishes with the appearance of a prevalent wider QRS-complex (usually wider than 120 ms; normal QRS: 0.06–0.10 s) followed by a prevalent T-wave (i.e., larger than usual). Therefore, it provides either shorter or larger RR-intervals (0.6 s< or >1.2 s) than normal (0.6–1.2 s) situations.

This phenomenon plays an important role in characterizing the PVC. Hence, our study exploited instantaneous RR-interval, using the Pan–Tompkins algorithm [51] to segment the PVC and the normal beats from the ECG signals. In this arrhythmia, the palpitations of the heart generate from ventricular Purkinje fibers despite the sinoatrial (SA) node [69]. However, the feature extraction of PVC is challenging to some extents.

In our proposed method, the pre-trained ResNet model extracts various levels of features from its input ECG beat images and achieved satisfactory results, as demonstrated in Table 5. It shows that the proposed method delivers better performance than anterior hand-crafted feature engineering techniques and deep learning methods. The method received overall accuracies of 99.74%, 99.89%, 99.93%, and 99.77% in T1, T2, T3, and T4, respectively. From the results of various evaluation metrics in the experiments, it is observed that the results for detecting PVC from the transformed beat images are almost the same, which indicates the generalization of our proposed method. Among the evaluation metrics, F1-score is more significant because it adopts the accuracy for a class imbalance dataset where recall (sensitivity) and precision are compiled, representing the method’s exactness and sensitivity at a time. Therefore, this metric usually provides poor results compared to other metrics. The overall F1-score is 99.18%, 99.75%, 99.84% and 99.64% in T1, T2, T3 and T4, respectively. The specificity of a method indicates the ability to correctly identify individuals who do not have a specific disease.

In our study, the average achieved specificities are 99.58%, 99.85%, 99.94% and 99.73% in T1, T2, T3 and T4, respectively. The method is also evaluated using hold-out and stratified k-fold strategies on both the datasets (MIT-BIH and INCART). The overall achieved accuracy, precision, recall, F1-score, and specificity on the MIT-BIH dataset using the hold-out evaluation technique are 97.90%, 95.30%, 95.10%, 95.20% and 94.30%, respectively. While the accuracy, precision, recall, F1-score, and specificity on INCART are 97.60%, 95.50%, 95.30%, 95.40%, and 94.60%, respectively.

On the other hand, the overall accuracy, precision, recall, F1-score and specificity on the MIT-BIH dataset using the stratified 10-fold evaluation technique are 98.50%, 97.20%, 97.05%, 97.13%, and 98.30%, respectively. While the accuracy, precision, recall, F1-score, and specificity on INCART are 98.55%, 97.80%, 97.70%, 97.75%, and 98.40%, respectively. The results on both datasets are almost similar by using the same strategy. However, the stratified technique provides better results than the hold-out technique. This is mainly due to less evaluating effects of the stratified strategy’s varying training and testing samples ratio. However, the experiments (T1, T2, T3, and T4) in patient-specific strategy are comparatively better than those of both the evaluation techniques. This evidences that the patient-specific strategy is more suitable for real-life applications than hold-out and stratified techniques.

From the literature survey of recent advancements of this domain, as summarized in Table 5, it is found that (i) features engineering-based works are significantly more than deep learning approaches, (ii) RR-interval is a more useful feature and (iii) the performance from the morphological based methods is usually better in comparison with the deep learning approaches. While the feature engineering-based methods have provided comparatively more satisfactory results, they have their shortcomings, such as (i) high professional knowledge is required for designing the algorithms of extracting features from ECG signals, (ii) extracted features are varied and prone to biases, (iii) accurate positioning of various waves such as P, Q, R, S, T as well as QRS-complex is challenging by a morphological based algorithm for extracting a PVC beat from the ECG signals correctly [75], (iv) these methods usually tend to over-fitting [76], mainly when they deal with big-data and (v) burdensome to deal with the data imbalance problem [37]. However, most deep learning methods can resolve these shortcomings. These methods have three main characteristics: (i) features are extracted automatically, so high professional knowledge is not needed, (ii) they are continuously optimized during the training for selecting the best features and removing non-redundancy, and (iii) they can easily handle the data imbalance problem. Hence, deep learning-based methods usually provide better results than morphological-based methods.

From Table 5, it is also observed that our proposed 2D CNN-based deep learning method delivers better performance when compared with the morphological-based techniques and the existing deep learning methods concerning identifying the PVCs from ECG signals. This is due to (i) transformation of segmented PVC beats into their corresponding images that reduce the strict time alignment requirement of beats during features extraction and (ii) implementation of several other techniques such as early stopping [48], weighted random sampler [49], Adam optimizer [50], on-the-fly augmentation [53] and stratified evaluation strategy, (iii) CNN’s learn the prevalence features from the signals, (iv) application of effective R-peak detection algorithm [51] to segment the PVC beats, (v) using of pre-trained model, which transfers the learning experiences from large datasets into small target dataset [77], (vi) ResNets’ effectively handle the over-fitting problems compared to AlexNets, VGGNets, GooLeNets, etc., and (vii) using of patient-specific mechanism. The patient-specific mechanism is more persuasive and fairer regarding real-life applications and more realistic in clinical scenarios. Furthermore, our chosen datasets are significantly imbalanced, where N beats play a dominating role in most of the recordings. Therefore, hold-out and k-fold validation strategies are not very suitable for evaluating the performance of the proposed method.

In this study, we have experimented and analyzed the performance of our proposed method only for PVC detection. However, it could also be employed to recognize other categories of arrhythmias from ECG signals. The proposed method could also be further applied in other electrophysiological signals such as electroencephalogram (EEG) [78,79], electromyogram (EMG) [80], electrooculogram (EOG) and Electrocorticography (ECoG) to detect the different categories of diseases from the human body.

## 4. Conclusions and Future Directions

The decision-making process still faces a substantial hurdle when manually identifying PVCs from cardiac rhythms. The manual method takes a long time and is unreliable. Because of this, automatic PVC detection using deep learning has great promise. While much research has been performed on morphological approaches for automatic PVC detection, these approaches have scalability issues, particularly with the ever-increasing data volume. Our research has shown that deep learning approaches can overcome the problem of morphological-based approaches. Due to the substantial intra-patient variability, real-world noises, and a lack of data volume for deep model training in practical applications, patient-specific and transfer learning processes now hold a great potential. On the other hand, transformation of time-series data into the corresponding images reduces the strict time alignment requirement when extracting the features as well as provides better visual perception in some scenarios. Hence, 2D based recognition approaches are more promising nowadays compared to 1D CNN. So, we have proposed a deep learning method based on 2D CNN using patient-specific and transfer learning mechanisms on two publicly available imbalanced datasets. As a result, the findings with the proposed method provide better effectiveness than the state-of-the-art hand-crafted and deep learning methods to identify the PVCs from ECGs. The outcomes also indicate that the performance of the proposed method is nearly unchanged despite the use of diverse strategies in numerous trials with two different heterogeneous datasets (they have different features) which demonstrates the method has good scalability and applicability. Therefore, it may be a helpful tool for cardiologists’ clinical decision support systems when using an offline or online approach.

The study implemented the ECG signal from a single lead, which could be enriched to study signals from multiple leads. Future research will examine signals from multiple leads to further enhance the experimental instances. Additionally, we have also planned to work on a number of interesting directions such as expanding the range of potential adaptation of the proposed method to the datasets of EEG, EMG signals for identifying other categories of human diseases such as epilepsy, Alzheimer’s, sleep apnea. A lightweight deep model narrates good efficiency for deploying one in real-life applications. Hence, we also intend to create a lightweight modified ResNet model for real-world applications that will make use of the micro-attention mechanism.

## Figures and Tables

**Table 1:** Confusion matrixes for all the experiments using the proposed method.

T1				T2				T3				T4			
	PL				PL				PL				PL		
TL		N	PVC	TL		N	PVC	TL		N	PVC	TL		N	PVC
	N	105,999	240		N	44,060	43		N	44,070	33		N	33,556	66
	PVC	62	9925		PVC	13	6410		PVC	04	6419		PVC	29	8287

TL: True Level; PL: Predicted Level.

**Table 2:** A summary of achieved metrics in all the experiments.

Experiments	Accuracy	Precision	Recall	F1-Score
T1	0.9974	0.9764	0.9938	0.9918
T2	0.9989	0.9933	0.9980	0.9975
T3	0.9993	0.9949	0.9993	0.9984
T4	0.9977	0.9921	0.9965	0.9964

**Table 3:** A summary of achieved metrics in all the experiments from the confusion matrix.

Exp.	Accuracy (%)		Precision (%)		Recall (%)		F1-Score (%)		Specificity (%)	
T1	N	99.74	N	99.94	N	99.77	N	99.86	N	99.38
	PVC	99.74	PVC	97.64	PVC	99.38	PVC	98.51	PVC	99.77
Average	99.74		98.79		99.58		99.19		99.58	
T2	N	99.89	N	99.94	N	99.90	N	99.92	N	99.80
	PVC	99.89	PVC	99.33	PVC	99.80	PVC	99.57	PVC	99.90
Average	99.89		99.64		99.85		99.75		99.85	
T3	N	99.93	N	99.99	N	99.93	N	99.96	N	99.94
	PVC	99.93	PVC	99.49	PVC	99.94	PVC	99.72	PVC	99.93
Average	99.93		99.74		99.94		99.84		99.94	
T4	N	99.77	N	99.82	N	99.80	N	99.81	N	99.65
	PVC	99.77	PVC	99.21	PVC	99.65	PVC	99.43	PVC	99.80
Average	99.77		99.51		99.73		99.62		99.73	

**Table 4:** The physiologic features of a normal ECG signal.

Phase	Description	Duration/Amplitude
P	The first upwards wave of the ECG	<80 ms
RR	The time interval between RR peaks	0.6–1.2 s
PR	The time between the P and the R wave	120–200 ms
QRS	The time between Q and S beats	80–120 ms
ST	The time between S and T beats	320 ms

**Table 5:** Various recent methods for identifying PVC.

Classifier Type/Approach	Features	Acc. (%)	Spec. (%)	Pre. (%)	Rec. (%)	F1-Score (%)
2D CNN (Proposed-T1) 2D CNN (Proposed-T2) 2D CNN (Proposed-T3) 2D CNN (Proposed-T4)	Transformation of time-series ECG data/signal into the respective 2D beat images	99.74 99.89 99.93 99.77	99.58 99.85 99.94 99.73	97.64 99.33 99.49 99.21	99.38 99.80 99.93 99.65	99.18 99.75 99.84 99.64
2D CNN [70]	Time frequency images	97.89	97.17	98.58	---	---
2D CNN [71]	Wavelet power spectrums	97.96	99.11	82.60	---	---
2D CNN [72]	Wavelet fusion method, Tucker-decomposition	90.84	99.86	78.60	---	---
DNN [73]	R-peak amplitude, S-peak amplitude, R-R interval time, Q-peak amplitude, ventricular activation time, QRS duration time	99.41	---	96.08	---	---
DNN [6]	Seven statistical and three morphological features	98.60	---	98.70	---	---
Adaptive Thresholding [74]	Energy wavelet coefficients	---	99.94	99.18	---	---
Artificial Immune Systems (AIS) [7]	Geometrical features	98.04	98.65	91.08	---	---
SVM [8]	Extraction of six features with several methodologies	99.78	99.37	99.91	---	---

## Data Availability

The data used in this study are mentioned in the manuscript and online available as an open access.
